# Phenotypic comorbidity network analysis of nasopharyngeal carcinoma: a multicenter study based on hospital discharge records

**DOI:** 10.3389/fonc.2026.1895769

**Published:** 2026-09-10

**Authors:** Yanqun Huang, Yifan Xu, Jiangnian Gong, Hao Ren, Lixuan Huang

**Affiliations:** 1 Department of Medical Equipment, The First Affiliated Hospital of Guangxi Medical University, Nanning, Guangxi, China; 2 Department of Radiology, The First Affiliated Hospital of Guangxi Medical University, Nanning, Guangxi, China; 3 Department of Radiology, The Second Affiliated Hospital of Guangxi Medical University, Nanning, Guangxi, China

**Keywords:** nasopharyngeal carcinoma, comorbidity analysis, network construction, phenotypic comorbidity, hospital discharge records

## Abstract

**Background:**

Nasopharyngeal carcinoma (NPC) patients frequently have multiple comorbidities that affect treatment efficacy and survival. Prior studies focused only on individual common comorbidities, lacking a comprehensive analysis of the full spectrum of chronic diseases in NPC patients from endemic areas. This study adopted phenotypic comorbidity networks (PCNs) based on large-scale hospital discharge data to systematically characterize the overall comorbidity patterns of endemic NPC patients.

**Methods:**

This retrospective cohort study was conducted using data from NPC patients identified in the electronic medical record systems of two large general hospitals in endemic regions between January 2010 and December 2023. Diagnoses were classified using ICD-10 (International Classification of Diseases, 10th Revision) codes at the 3-digit level. PCNs were constructed to represent comorbidity patterns, where nodes represented comorbidities, and edges depicted significant associations. Subgroup analysis was conducted by sex and metastatic status.

**Results:**

A total of 18481 NPC patients were involved in the study. NPC patients’ median age was 47–48 years. In Datasets 1 and 2, 82.9% and 78.2% of patients, respectively, had fewer than three comorbidities. Hypertension, chronic sinusitis, other anemias and disorders of fluid, electrolyte and acid-base balance were the top 4 comorbidities with the highest prevalence in all subgroups. The average number of comorbidities was higher in males than in females and in the non-metastatic subgroup compared with the metastatic subgroup. PCNs in males and non-metastatic patients exhibited more complex interconnections. Significant gender disparities existed in multiple comorbidities. Chronic viral hepatitis and kidney-ureteral calculi predominated in males, while fluid, electrolyte and acid-base disturbances along with nontoxic goiter were more prevalent in females. The PCN of the aplastic anemia subgroup was more complex than those of the two secondary malignant neoplasm subgroups.

**Conclusions:**

This study revealed the specific characteristics in comorbidity patterns of NPC patients. There were disparities in the prevalence of individual comorbidities and their complex coexistence among NPC patients as stratified by sex and metastatic status. These findings highlight the importance of understanding comorbidity patterns and their interactions in NPC patients, which are noteworthy for guiding personalized medical treatments and developing comprehensive strategies for disease management and prognosis improvement.

## 1 Introduction

Nasopharyngeal carcinoma (NPC) is one of the most common malignant tumors of the head and neck (1), characterized by distinct ethnic and geographic distribution patterns. It has a particularly high incidence in South China and Southeast Asia, notably in Guangxi and Guangdong (2). The standardized incidence was as high as 20-30/100000 (3). The surgical treatment of NPC mainly fails to achieve satisfactory outcomes. However, NPC tissues exhibit high sensitivity to radiation, making comprehensive treatment based on radiotherapy and chemotherapy the preferred approach for achieving radical cure (4).

Comorbidities refer to the coexistence of more than one distinct disease in an individual, in addition to the primary disease under consideration (5). Typically, an index disease is specified, and any additional conditions are classified as comorbidities. Previous research has demonstrated that 75% of cancer patients have at least one comorbidity (6). NPC frequently occurs in older patients and is associated with a higher prevalence of comorbidities due to age-related physiological decline and functional impairment. A recent study revealed that recurrence with metastasis (48%) and internal medical disease unrelated to NPC (32%) are the primary causes of death in elderly NPC patients (7). Previous studies investigating comorbidities in elderly patients with NPC have demonstrated that hypertension, chronic lung diseases, fatty liver disease, and diabetes mellitus are the most prevalent comorbidities within this population (8). In addition, growing evidence highlights that the presence of multiple comorbidities significantly influences clinical outcomes and quality of life in NPC patients. Comorbidities have been independently associated with poorer overall survival (OS) (8–10), as demonstrated in studies where comorbidity emerged as a significant prognostic factor for OS in NPC patients (hazard ratio (HR)=2.027) (11). Therefore, enhancing understanding and attention to the disease patterns that coexist with NPC is essential for effective disease management. This includes promoting multidisciplinary collaborative treatment, implementing personalized medical approaches, and advancing scientific disease management strategies.

In recent years, the study of disease comorbidity has remarkably attracted scholars’ attention both from molecular network mechanisms and clinical observations. Previous research on concomitant diseases in NPC patients has mainly concentrated on specific conditions, such as radiation-induced parotid gland injury and radiation-induced encephalopathy (12). Although certain comorbidities, such as hypertension and diabetes, are well-recognized (13), many others are likely to remain unidentified. The overall pattern of comorbidities in NPC is still poorly understood. A new study demonstrated the potential comorbidity patterns in elderly NPC patients, emphasizing the importance of examining complications to enhance treatment strategies and the comprehensive management of this population (8). However, to date, no study concentrated on NPC patients’ comorbidity patterns across all age groups in endemic regions.

Recently, phenome, which was the comprehensive set of an organism’s observable traits shaped by genetic and environmental interactions, has emerged as a critical framework for understanding complex diseases (14). The past decade has witnessed an explosion of patient health data, particularly through electronic health records, enabling the extraction of latent comorbidity information from clinical histories and providing a valuable resource for evaluating the entire diagnostic spectrum in comorbidity research. These records, including a large amount of health information in the form of International Classification of Diseases, Tenth Revision (ICD-10), hold significant potential for enhancing the understanding of comorbidity patterns (15). Notably, the hierarchical layout of ICD-10 diagnostic codes serves as a valuable resource for mapping multidimensional comorbidity networks.

The recent advancement of network theory has introduced a novel approach to understanding the intricate interrelations among diseases (16). The phenotypic comorbidity networks (PCNs), also referred to as the disease co-occurrence network, has been extensively utilized to study comorbidity patterns in various chronic diseases, such as depression (17), lung cancer (18), ischemic heart disease (19), osteoporosis (20), hepatocellular carcinoma (21), colorectal cancer (22), and chronic obstructive pulmonary disease (COPD) (23). Compared with traditional comorbidity analysis methods, PCN provides a new perspective to understand the comorbidity phenomenon. The network theory provides a mathematical language and analytical tools for understanding the structure and dynamics of PCNs, while systems biology approaches inject real biological content and data into PCNs. The combination of the two makes PCNs a powerful tool to explore the complex face of diseases at the network level, obtain a deeper understanding of the mechanism of disease occurrence and development, and then develop more effective and personalized disease prevention and treatment strategies.

The present study aimed to systematically analyze all comorbid diseases using a PCN based on large-scale hospital discharge records, and to present a comprehensive comorbidity pattern among NPC patients in endemic regions, providing valuable theoretical insights to inform current treatment practices and comprehensive management of comorbidity in NPC patients.

## 2 Materials and methods

### 2.1 Study population

**Figure 1:**
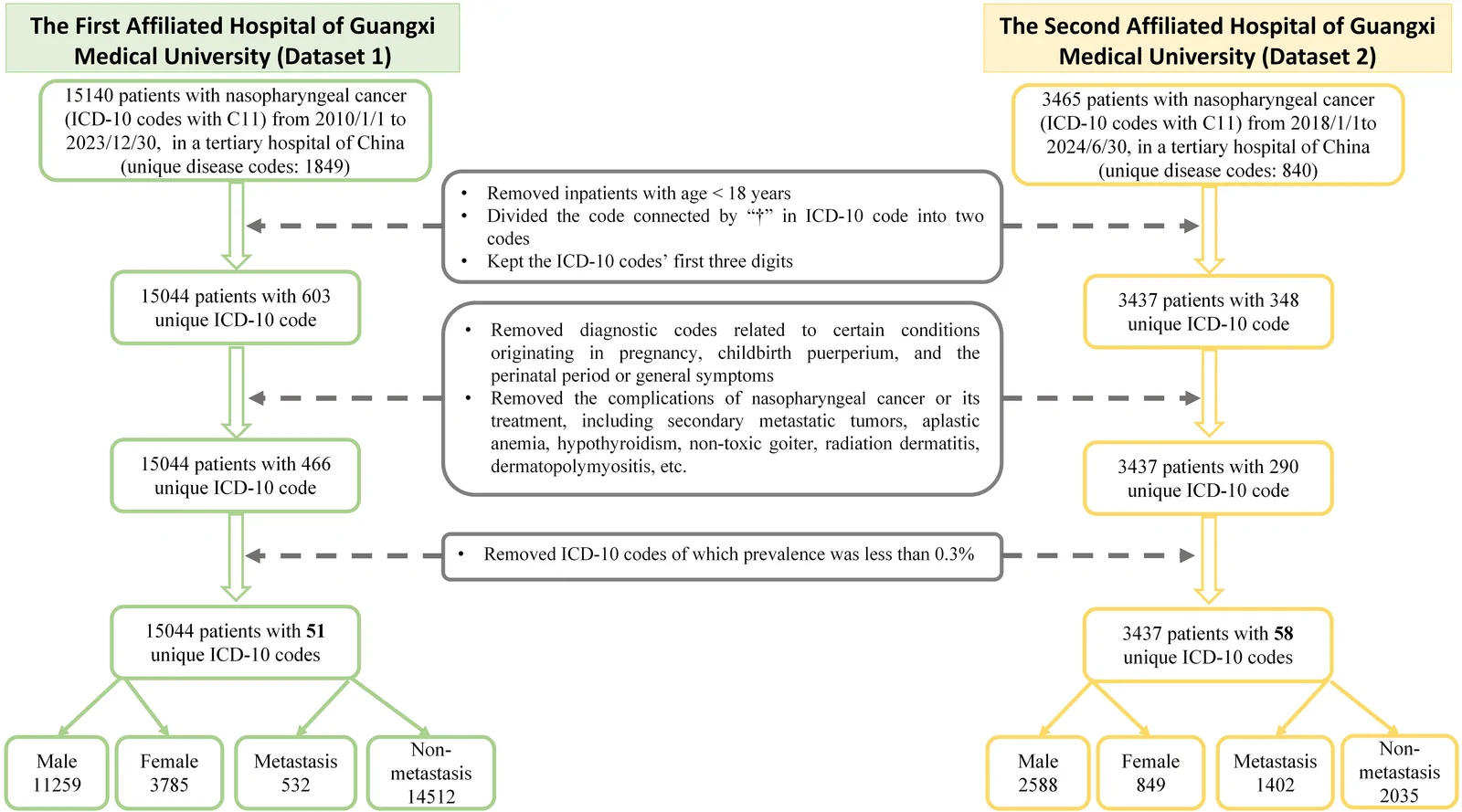
Study participants’ enrollment flowchart.

A retrospective cohort study was performed using discharge data of patients from two tertiary hospitals in an NPC-endemic area of China to conduct internal repeated validation across hospital cohorts (**Figure 1**). This study was approved by the Medical Ethics Committee of the First Affiliated Hospital of Guangxi Medical University (approval number 2024-E682-01). For clarity, the datasets were labelled Dataset 1 and Dataset 2. Patient information (including age, sex and diagnoses) was extracted from electronic medical record (EMR) systems, with diagnoses coded according to ICD-10 codes. NPC cases were identified using the ICD-10 code C11. Dataset 1 and Dataset 2 contained 15,044 and 3,437 hospitalizations, corresponding to 50 and 57 distinct ICD-10 codes, respectively. Notably, to exclude the complications of NPC or its treatment, some chronic conditions that occurred in the index hospitalization were excluded, including secondary metastatic tumors, aplastic anemia, hypothyroidism, non-toxic goiter, radiation dermatitis, dermatopolymyositis, etc. (24). The study population was stratified by sex and tumor metastasis status. Metastasis in our stratification refers to metastatic lesions identified at the time of the first NPC diagnosis. Patients who developed metastasis in the later treatment and follow-up period were not classified into the metastatic subgroup. We then calculated the prevalence of comorbidities along with 95% confidence intervals (CIs).

### 2.2 Differences in comorbidity prevalence

To evaluate variations in comorbidity prevalence across sex and tumor metastasis status, we calculated relative prevalence differences and assessed statistical significance via the Z-test. For instance, for sex, the calculation was based on Equation 1:

(1) $$d=\frac{P_{male}-P_{female}}{(P_{male}+P_{female})/2}$$

where *P_male_* and *P_female_* denote the prevalence of comorbidities in males and females, respectively.

A difference in comorbidity prevalence was considered significant if the relative difference exceeded 0.1 and the absolute difference was statistically significant. We further analyzed comorbidities with significant prevalence differences. A comorbidity was defined as enriched when its prevalence increased by at least 0.5-fold. For example, a comorbidity was regarded as enriched in males if it showed a significant sex-based prevalence difference and its prevalence in males was at least 0.5-fold higher than that in females. Spearman correlation analysis was used to examine the association between age and comorbidity prevalence. We also applied K-means clustering to categorize comorbidities of NPC patients based on age-related trends in their prevalence. We clustered comorbidities (rather than patients) to better characterize the age-dependent evolution of these conditions among NPC patients.

### 2.3 PCN construction

A PCN was constructed to depict the coexistence of all comorbidities, where nodes (3-digit ICD-10 codes) were linked by edges. Node size reflected disease prevalence, and color indicated the ICD-10 disease category. The cosine index, which is unaffected by sample size, was used to quantify comorbidity strength by accounting for co-occurrence and prevalence. A cutoff value was defined to identify comorbidity coexistence using the cosine index via analyzing the correlation between the Pearson correlation coefficient and the cosine index. This approach ensured that both metrics yielded an equal number of statistically significant comorbid co-occurrences across the two networks. Equations 2 and 3 define the cosine index and Pearson correlation coefficient, respectively. Significance of $\emptyset_{ab}\neq 0$ was assessed using t-test (Equation 4).

(2) $$Cosine\;index=\frac{n_{ab}}{\sqrt{n_{a}*n_{b}}}$$

(3) $$\emptyset_{ab}=\frac{n_{ab}*N-n_{a}*n_{b}}{\sqrt{n_{a}*n_{b}*\left(N-n_{a}\right)*\left(N-n_{b}\right)}}$$

(4) $$t=\frac{\emptyset_{ab}*\sqrt{n-2}}{\sqrt{1-\emptyset_{ab}}},n=max\left(n_{a},n_{b}\right)$$

In Equations 2–4, N denotes the total number of NPC patients, and *n_a_*, *n_b_*, and *n_ab_* indicate the number of patients diagnosed with disease *a*, disease *b*, and both diseases, respectively.

### 2.4 PCN’s properties

The structural properties of the PCN were assessed using network indices, including density, degree, average degree of neighbors, and betweenness centrality. Density indicates network compactness, measured by the ratio of significant connections to potential connections, and a higher density represents more interconnected comorbidities. Degree signifies the number of linked comorbidities. The median and interquartile range (IQR) were used to describe the degree distribution of the PCN. Comorbidities directly connected to one another are referred to as neighbors. The average degree of neighbors was calculated to evaluate connectivity among them. Betweenness centrality measures a comorbidity’s centrality in the network as the number of shortest paths between any two other comorbidities that pass through it, divided by the total number of all possible paths between those comorbidities. A high betweenness centrality suggests that a comorbidity may serve as a bridge or endpoint connecting other comorbidities. To determine the most significant comorbidity within the PCN, the PageRank algorithm was employed, accounting for edge weights. A higher PageRank score indicates a greater impact on the network. The top ten comorbidities with the highest PageRank scores were identified as the most important nodes in the PCN.

Analyses and visualizations were conducted using R 4.3.1 and Python 3.7.

## 3 Results

### 3.1 Patients’ characteristics and comorbidity prevalence

NPC patients’ characteristics in the two datasets are presented in **Table 1**. In both datasets, approximately three-quarters (74.8% and 75.3% in Datasets 1 and 2, respectively) of patients were male. Patients’ mean age in the two datasets was similar, including 47 years in Dataset 1 and 48 years in Dataset 2. Additionally, males were generally older than females in both datasets. In Dataset 1, 3.5% of patients had metastatic NPC, whereas the corresponding proportion was 40.8% in Dataset 2. NPC patients carried a moderate comorbid burden, with an average number of 1.6 comorbidities in Dataset 1 and 1.8 comorbidities in Dataset 2. Notably, fewer than three comorbidities were identified in 82.9% and 78.2% of patients in Datasets 1 and 2.

**Table 1:** Nasopharyngeal cancer patients’ characteristics in different groups.

	Dataset 1					Dataset 2				
Demographic and clinical characteristics	Overall	Male subgroup	Female subgroup	Metastasis subgroup	Non-metastasis subgroup	Overall	Male subgroup	Female subgroup	Metastasis subgroup	Non-metastasis subgroup
	(N = 15044)	(N = 11259)	(N = 3785)	(N = 532)	(N = 14512)	(N = 3437)	(N = 2588)	(N = 849)	(N = 1402)	(N = 2035)
Male, n (%)	11259 (74.8)	/	/	436 (82.0)	10823 (74.6)	2588 (75.3)	/	/	1132 (80.7)	1456 (71.5)
Age (years), mean (SD)	47.1 (11.2)	47.4 (11.1)	46.0 (11.3)	48.6 (12.3)	47.0 (11.1)	48.6 (11.2)	48.9 (10.9)	47.7 (12.0)	48.2 (10.3)	48.8 (11.7)
Age group (years), n (%)										
18-39	3904 (26.0)	2774 (24.6)	1130 (29.9)	115 (21.6)	3789 (26.1)	785 (22.8)	554 (21.4)	231 (27.2)	327 (23.3)	458 (22.5)
40-49	4921 (32.7)	3756 (33.4)	1165 (30.8)	140 (26.3)	4781 (32.9)	882 (25.7)	639 (24.7)	243 (28.6)	331 (23.6)	551 (27.1)
50-59	4204 (27.9)	3162 (28.1)	1042 (27.5)	184 (34.6)	4020 (27.7)	1280 (37.2)	1050 (40.6)	230 (27.1)	607 (43.3)	673 (33.1)
60-69	1670 (11.1)	1286 (11.4)	384 (10.1)	70 (13.2)	1600 (11.0)	417 (12.1)	295 (11.4)	122 (14.4)	122 (8.7)	295 (14.5)
≥70	345 (2.3)	281 (2.5)	64 (1.7)	23 (4.3)	322 (2.2)	73 (2.1)	50 (1.9)	23 (2.7)	15 (1.1)	58 (2.9)
Number of comorbidities, mean (SD)	1.6 (0.9)	1.7 (1.0)	1.6 (0.9)	1.6 (0.9)	1.6 (0.9)	1.8 (0.9)	1.8 (0.9)	1.9 (0.9)	1.5 (0.7)	2.1 (1.0)
Number of comorbidities, n (%)										
1	9129 (60.7)	6694 (59.5)	2435 (64.3)	329 (61.8)	8800 (60.6)	1509 (43.9)	1163 (44.9)	346 (40.8)	900 (64.2)	609 (29.9)
2	3342 (22.2)	2516 (22.3)	826 (21.8)	109 (20.5)	3233 (22.3)	1178 (34.3)	874 (33.8)	304 (35.8)	381 (27.2)	797 (39.2)
3	1759 (11.7)	1413 (12.5)	346 (9.1)	66 (12.4)	1693 (11.7)	555 (16.1)	409 (15.8)	146 (17.2)	99 (7.1)	456 (22.4)
4	621 (4.1)	476 (4.2)	145 (3.8)	26 (4.9)	595 (4.1)	172 (5.0)	123 (4.8)	49 (5.8)	20 (1.4)	152 (7.5)
≥5	193 (1.3)	160 (1.4)	33 (0.9)	2 (0.4)	191 (1.3)	23 (0.7)	19 (0.7)	4 (0.5)	2 (0.1)	21 (1.0)

**Figure 2:**
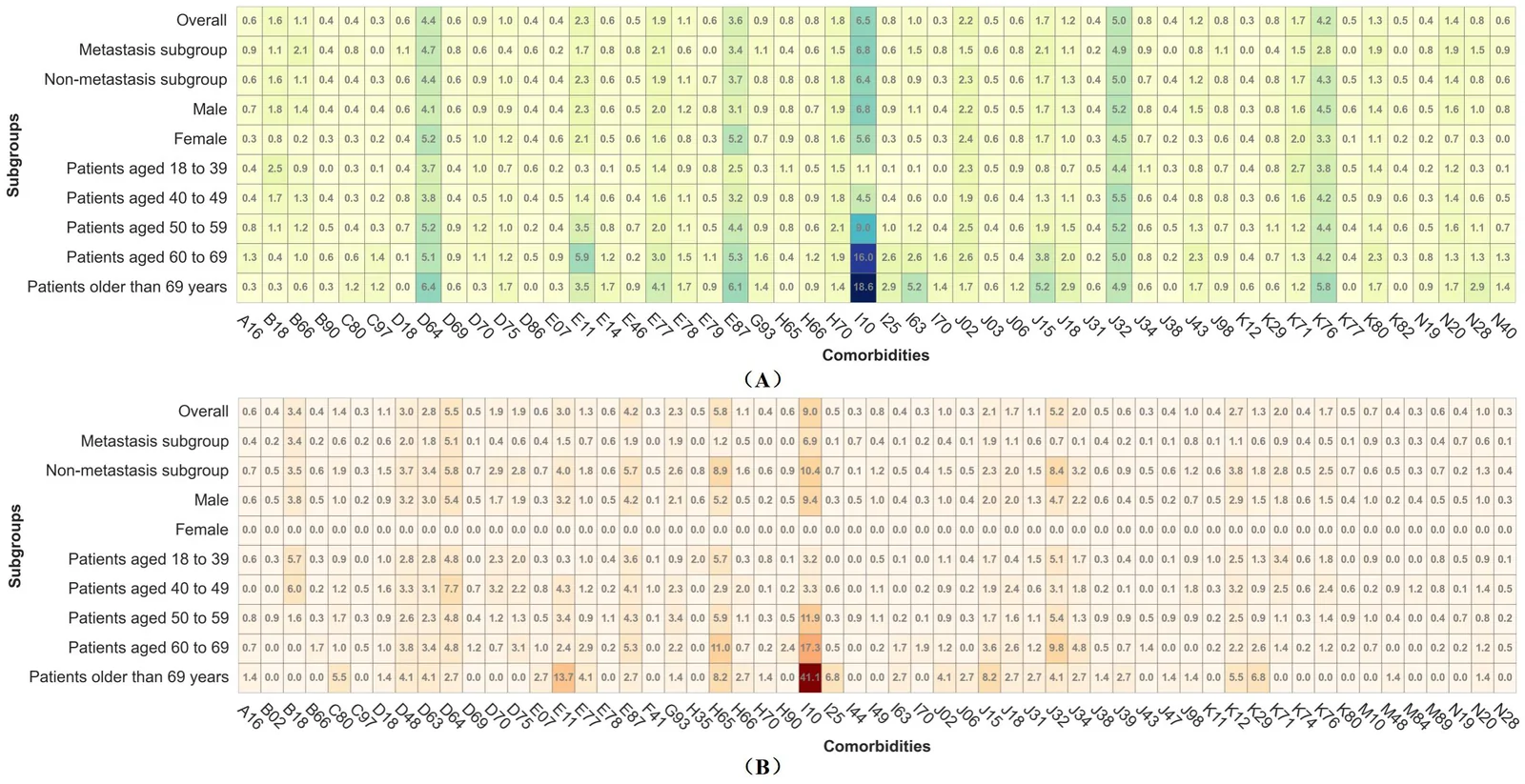
Prevalence of comorbidities across patient subgroups in Dataset 1 **(A)** and Dataset 2 **(B)**. Values are presented as prevalence (%), and darker colors correspond to higher prevalence rates.

The prevalence of comorbidities in various subgroups is presented in **Figure 2**. In both datasets, the top four most prevalent comorbidities in all subgroups were hypertension (I10), chronic sinusitis (J32), other anemias (D64) and disorders of fluid, electrolyte and acid-base balance (E87), with the overall prevalence rates of 6.5%, 5.0%, 4.4% and 3.6% in Dataset 1, and 9.00%, 5.2%, 5.5% and 4.2% in Dataset 2, respectively. Particularly, nonsuppurative otitis media (H65) was also common in all subgroups in Dataset 2, with the prevalence of 5.8%. Older patients tended to have a higher prevalence of hypertension (I10).

**Figure 3:**
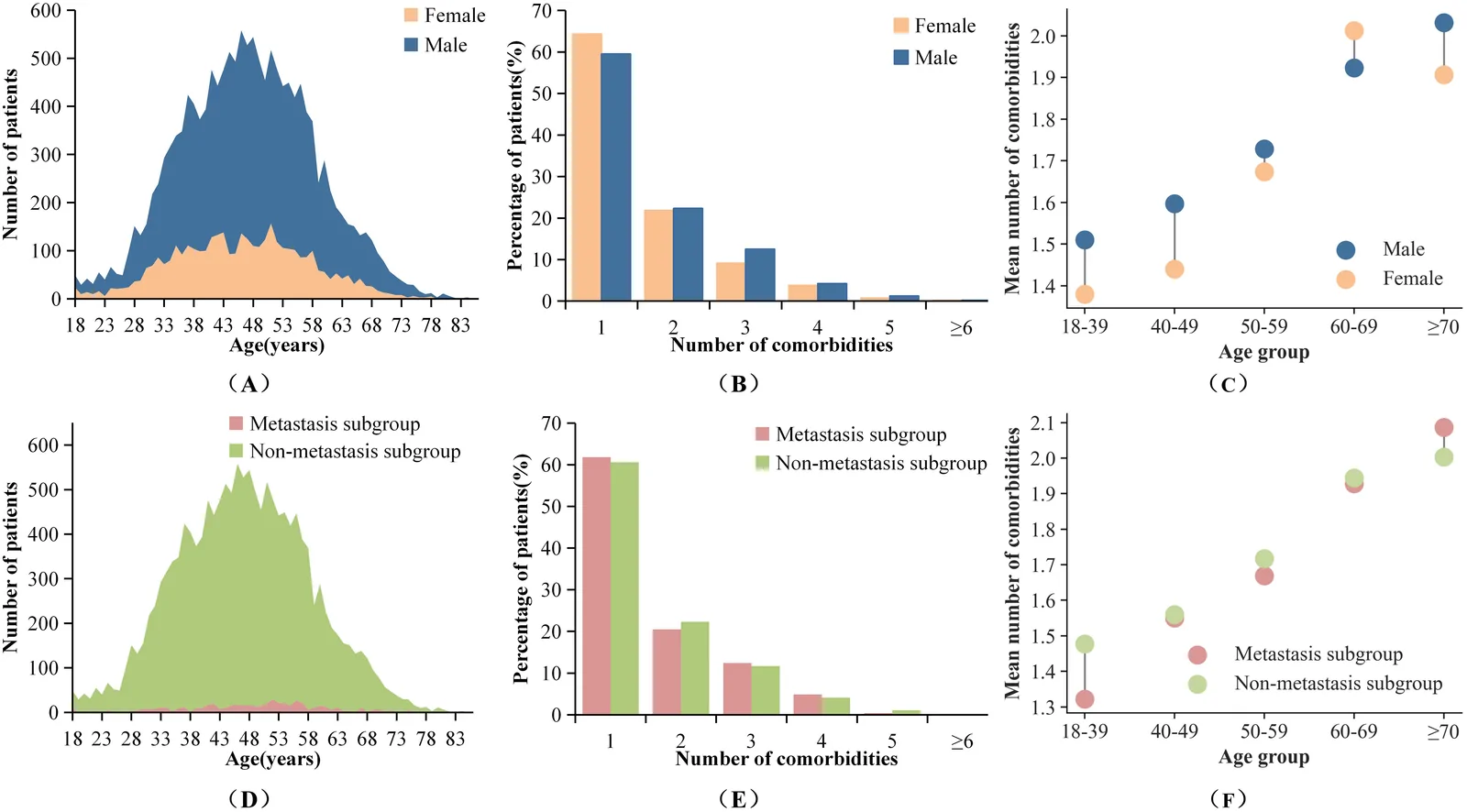
Age distribution **(A, D)**, comorbidity count distribution **(B, E)**, and mean comorbidity burden by age group **(C, F)** in nasopharyngeal cancer patients from Dataset 1, with **(A–C)** stratified by sex and **(D–F)** as their counterparts stratified by metastasis status.

Age distribution and comorbidity count across subgroups in Dataset 1 are shown in **Figure 3**. The average number of comorbidities was higher in males than in females, as well as in the non-metastasis subgroup relative to the metastasis subgroup (**Figure 3C, F**). In Dataset 1, among patients aged under 70 years, both males and females exhibited a rising mean number of comorbidities with advancing age (**Figure 3C**). Overall, patients in Dataset 1 presented an increasing number of comorbidities as age advanced, irrespective of metastasis status (**Figure 3F**). Patients without metastasis in Dataset 2 also demonstrated an upward trend in the mean comorbidity count with age (**Supplementary Figure 1** in **Supplementary Materials**).

### 3.2 Differences in comorbidity prevalence

Regarding subgroup variations, most comorbidities were shared across all subgroups, whereas a small number were unique to specific subgroups and showed relatively low prevalence (**Table 2**). Several comorbidities exhibited significant differences in prevalence between metastatic and non-metastatic subgroups, all showing higher prevalence in the non-metastasis subgroup. For instance, malignant neoplasms of primary multiple sites (C97), diseases of the vocal cords and larynx (J38), and stomatitis and related lesions (K12) were more common in the non-metastasis subgroup, with prevalence rates of 0.3%, 0.4%, and 0.3%, respectively. Similarly, disorders of purine and pyrimidine metabolism (E79), liver disorders in diseases classified elsewhere (K77), and other diseases of gallbladder (K82) also presented lower prevalence in the metastasis subgroup, with a subgroup difference value of -0.7, -0.5, and -0.5, while other diseases of liver (K76) showed the most prominent metastasis-related difference at -1.5. Among comorbidities with statistically significant gender-based prevalence differences, obvious disparities were observed between sexes. Specifically, chronic viral hepatitis (B18) and calculus of kidney and ureter (N20) were more common in males, whereas other disorders of fluid, electrolyte and acid-base balance (E87) were markedly more prevalent in females.

**Table 2:** Absolute prevalence differences of enriched comorbidities across subgroups in Dataset 1.

ICD-10 codes	Disease names	Overall (95% CI)	Male-female (95% CI)	Metastasis-non-metastasis (95% CI)
A16	Respiratory tuberculosis	0.6 (0.5, 0.7)	0.4 (0.2, 0.6)	–
B18	Chronic viral hepatitis	1.6 (1.4, 1.8)	1.0 (0.6, 1.4)	–
B66	Other fluke infections	1.1 (0.9, 1.3)	1.2 (0.9, 1.5)	–
C97	Malignant neoplasms of primary multiple sites	0.3 (0.2, 0.4)	–	-0.3 (-0.4, -0.2)
E79	Disorders of purine and pyrimidine metabolism	0.6 (0.5, 0.8)	0.5 (0.3, 0.7)	-0.7 (-0.8, -0.6)
E87	Other disorders of fluid, electrolyte and acid-base balance	3.6 (3.3, 3.9)	-2.1 (-2.9, -1.3)	–
I25	Chronic ischemic heart disease	0.8 (0.6, 0.9)	0.6 (0.4, 0.8)	–
I63	Cerebral infarction	1.0 (0.8, 1.1)	0.6 (0.3, 0.9)	–
J38	Diseases of vocal cords and larynx, not elsewhere classified	0.4 (0.3, 0.5)	–	-0.4 (-0.5, -0.3)
J43	Emphysema	1.2 (1.0, 1.3)	1.2 (0.9, 1.5)	–
K12	Stomatitis and related lesions	0.3 (0.2, 0.4)	–	-0.4 (-0.5, -0.3)
K76	Other diseases of liver	4.2 (3.9, 4.5)	–	-1.5 (-2.9, -0.1)
K77	Liver disorders in diseases classified elsewhere	0.5 (0.4, 0.6)	0.5 (0.3, 0.7)	-0.5 (-0.6, -0.4)
K82	Other diseases of gallbladder	0.5 (0.4, 0.6)	0.4 (0.2, 0.6)	-0.5 (-0.6, -0.4)
N19	Unspecified kidney failure	0.4 (0.3, 0.5)	0.3 (0.1, 0.5)	–
N20	Calculus of kidney and ureter	1.4 (1.2, 1.6)	0.9 (0.5, 1.3)	–
N28	Other disorders of kidney and ureter, not elsewhere classified	0.8 (0.7, 1.0)	0.7 (0.4, 1.0)	–
N40	Hyperplasia of prostate	0.6 (0.5, 0.7)	0.8 (0.6, 1.0)	–

**Figure 4:**
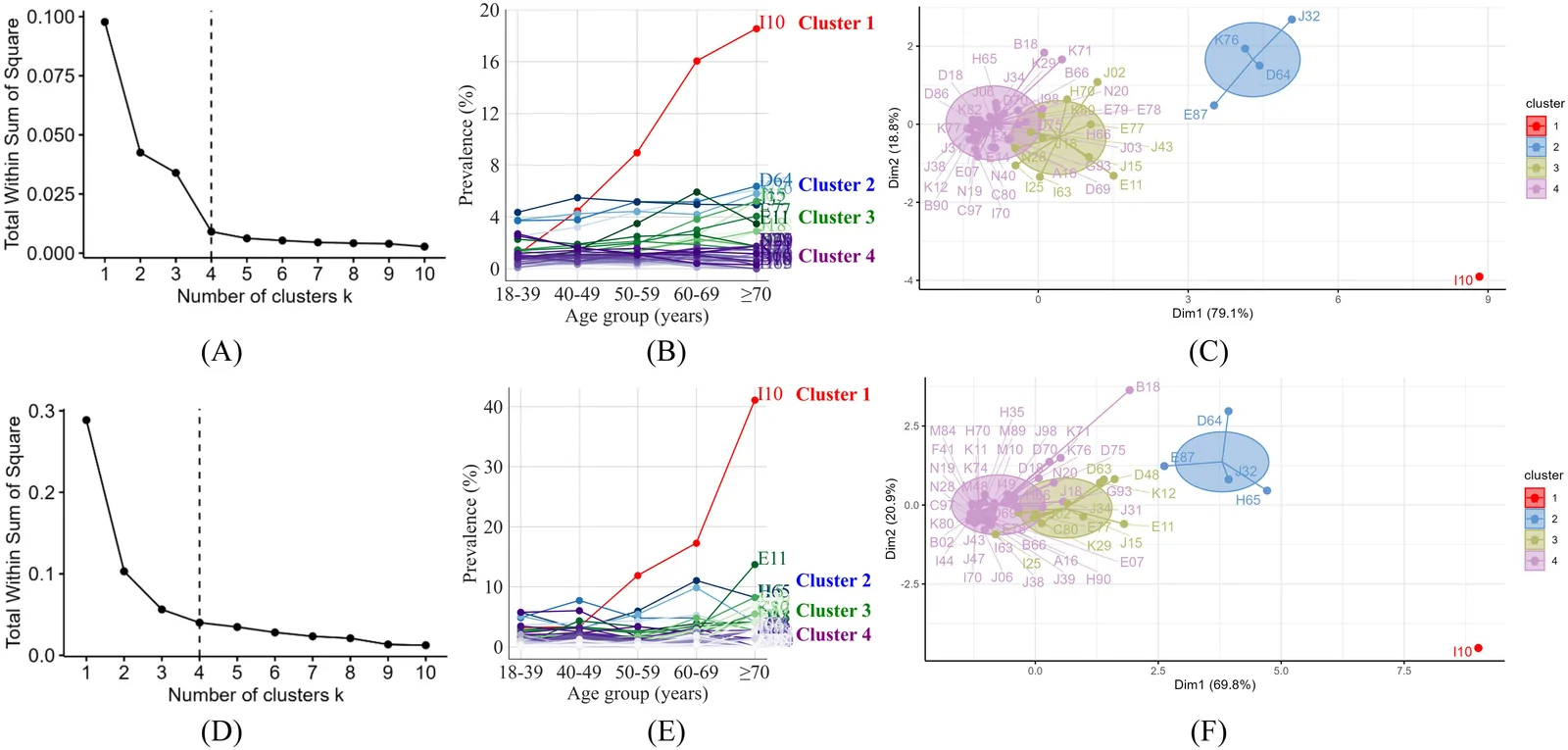
Clusters of comorbidities in patients with nasopharyngeal carcinoma based on age-specific prevalence. **(A, D)** Optimal number of clusters determined using the K-means clustering algorithm. **(B, E)** Age-specific prevalence of comorbidities in each cluster. **(C, F)** Clustering plots. Based on the principal component analysis, comorbidity prevalence across five age dimensions (18-39, 40-49, 50-59, 60-69, and ≥70 years) was reduced to 2 dimensions (x-lab and y-lab). **(A–C)** were related to Dataset 1. **(D–F)** were the counterparts of **(A–C)** in Dataset 2. The ICD-10 codes are detailed in **Supplementary Materials** **Supplementary Table 1**.

Cluster analysis of comorbidities (excluding NPC, ICD-10 C11) identified 4 optimal clusters for both datasets via elbow curves, with consistent grouping patterns (**Figure 4**). Dataset 1 contained 1, 4, 11, 34 comorbidities in Clusters 1-4, and Dataset 2 had 1, 4, 11, 41 respectively. Cluster 1 only included hypertension (I10), whose prevalence rose sharply with age and peaked at 18.6% (Dataset 1) and over 41.1% (Dataset 2) in patients aged ≥70 years, and this condition acted as an isolated outlier in the dimensionality-reduction biplots (**Figures 4C, F**). Cluster 2 comprised four moderate-prevalence diseases with age-increasing prevalence that peaked in the oldest age group; the shared conditions were other anemias (D64), chronic sinusitis (J32) and fluid-electrolyte disorders (E87), while Dataset 1 additionally included other liver diseases (K76) and Dataset 2 contained nonsuppurative otitis media (H65), forming an independent elliptical cluster in biplots. Cluster 3 covered 11 intermediate-prevalence disorders with mild age-dependent prevalence growth, with shared representative diseases including type 2 diabetes mellitus (E11), glycoprotein metabolism disorders (E77) and chronic ischemic heart disease (I25). Cluster 4 was the largest subgroup featuring persistently low prevalence across all age strata, exemplified by chronic viral hepatitis (B18) and unspecified kidney failure (N19), and these disorders clustered closely with Cluster 3 in the two-dimensional plots.

### 3.3 Overall PCN

**Figure 5:**
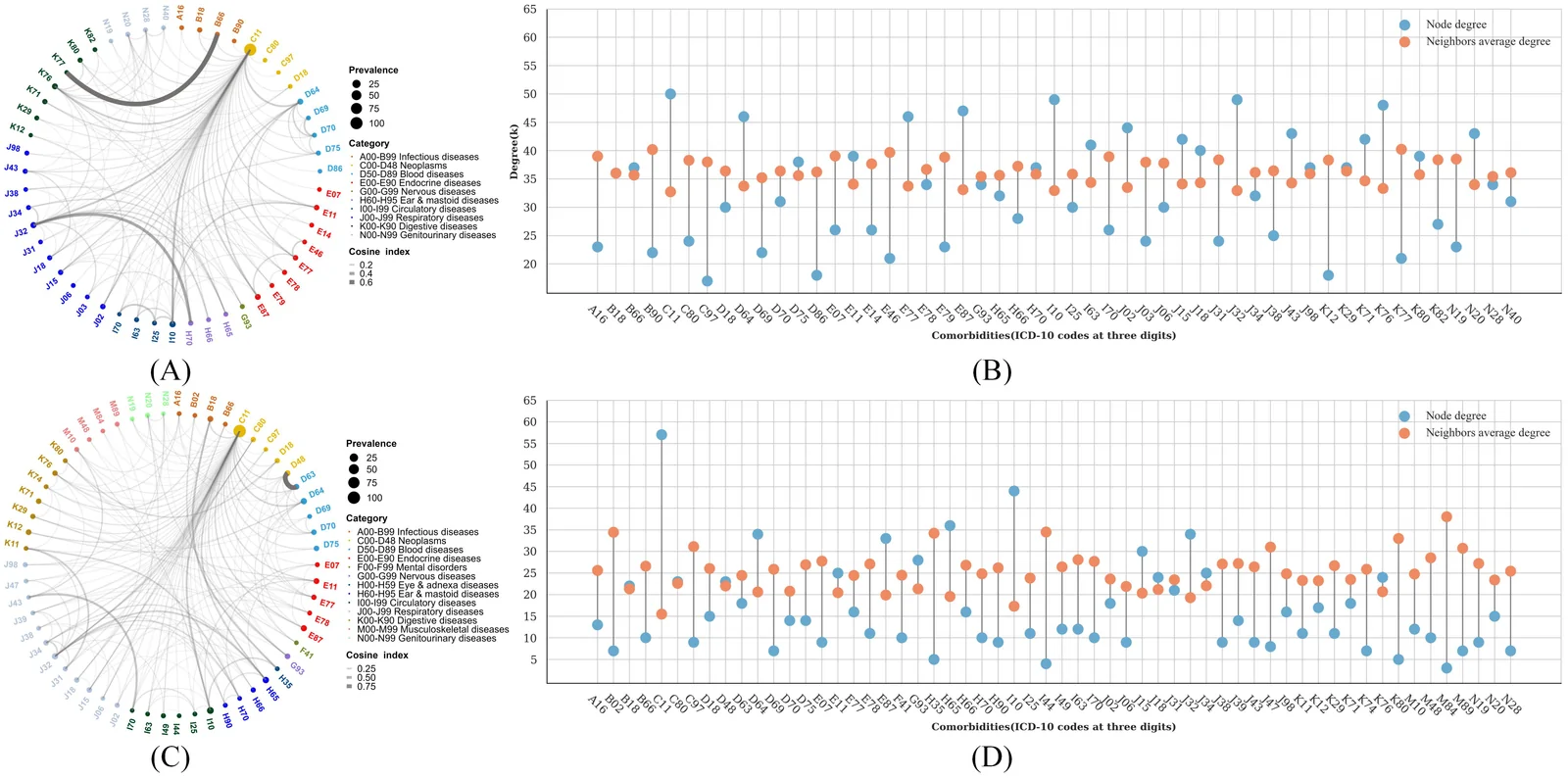
Phenotypic comorbidity networks (PCNs) for patients with nasopharyngeal cancer **(A, C)**, along with the degree distributions of comorbidities in the PCNs **(B, D)**. **(A, B)** belong to Dataset 1, while **(C, D)** belong to Dataset 2. All diseases and ICD-10 codes are detailed in **Supplementary Materials** (**Supplementary Table 1**).

The PCNs of NPC patients in Dataset 1 and Dataset 2 contained 140 and 177 comorbid disease pairs with a cosine index >0.05, respectively (**Figure 5**). Hypertension (I10), chronic sinusitis (J32) and other anemias (D64) were core high-prevalence hub conditions in both networks, and their strong inter-disease connections substantially increased multimorbidity complexity. Respiratory diseases (ICD-10 J codes) acted as the primary mediators of comorbidity linkages across both cohorts, with 11 distinct respiratory disorders in Dataset 1 and 12 in Dataset 2. In Dataset 1, the key mediator categories shaping network connectivity were 11 respiratory diseases (J00-J99), 5 blood diseases (D50-D89), and 4 malignant neoplasms (C00-D48), and these three disease groups remained highly prevalent and structurally central within the PCN of Dataset 2.

Network properties of PCNs stratified by sex, metastasis status, and age group across Dataset 1 and Dataset 2 are shown in **Table 3**. Male and non-metastatic NPC patients had more complex comorbidity connections. In Dataset 1, metastatic patients had fewer comorbidity nodes (44) and much lower network density (0.149), median degree (4.0) and median neighbor degree (16.0) than non-metastatic patients (50 nodes, density 0.653, median degree 32.0, median neighbor degree 35.7), suggesting weaker comorbidity links. This pattern held in Dataset 2. Males had higher network metrics than females in both cohorts, e.g., Dataset 1 density reached 0.614 in males versus 0.320 in females. Network complexity peaked at age 50–59 years (Dataset 1 density 0.419) and declined with age, with patients ≥70 years showing the sparsest comorbidity networks.

**Table 3:** Phenotypic comorbidity network’s properties in nasopharyngeal cancer patients. .

Subgroup	Dataset 1				Dataset 2			
	Nodes, n	Density	Degree, median (IQR)	Degree of neighbors, median (IQR)	Nodes, n	Density	Degree, median (IQR)	Degree of neighbors, median (IQR)
All	50	0.661	32.0 (24.2-39.8)	36.0 (34.5-38.0)	57	0.284	12.0 (9.0-21.0)	24.8 (22.0-27.1)
Sex								
Female	49	0.32	12.0 (9.0-18.0)	24.1 (20.9-25.8)	50	0.175	7.0 (4.0-9.8)	18.3 (15.6-21.7)
Male	50	0.614	30.5 (21.2-37.8)	34.0 (33.1-36.3)	57	0.232	10.0 (7.0-16.0)	22.8 (19.7-25.0)
Metastasis status								
Metastasis	44	0.149	4.0 (3.0-7.2)	16.0 (13.4-19.5)	53	0.11	4.0 (3.0-6.0)	19.5 (16.2-27.7)
Non-metastasis	50	0.653	32.0 (24.2-39.8)	35.7 (34.2-37.6)	57	0.264	12.0 (8.0-18.0)	23.7 (21.0-25.8)
Age group (years)								
18-39	49	0.277	12.0 (7.0-17.0)	22.1 (19.2-24.3)	48	0.132	5.0 (3.0-7.0)	17.6 (14.8-23.5)
40-49	50	0.377	18.0 (11.2-22.8)	25.1 (23.5-27.0)	51	0.137	5.0 (3.0-9.0)	18.7 (15.3-23.2)
50-59	50	0.419	18.0 (13.2-26.8)	27.3 (25.6-29.6)	55	0.165	6.0 (5.0-10.0)	20.4 (17.7-23.9)
60-69	50	0.316	13.0 (8.2-20.5)	24.3 (21.8-27.3)	47	0.151	6.0 (3.0-7.5)	18.3 (15.3-23.5)
≥70	44	0.176	5.0 (4.0-9.0)	18.8 (14.8-23.2)	32	0.152	3.0 (2.0-5.2)	14.0 (11.2-17.6)

**Figure 6:**
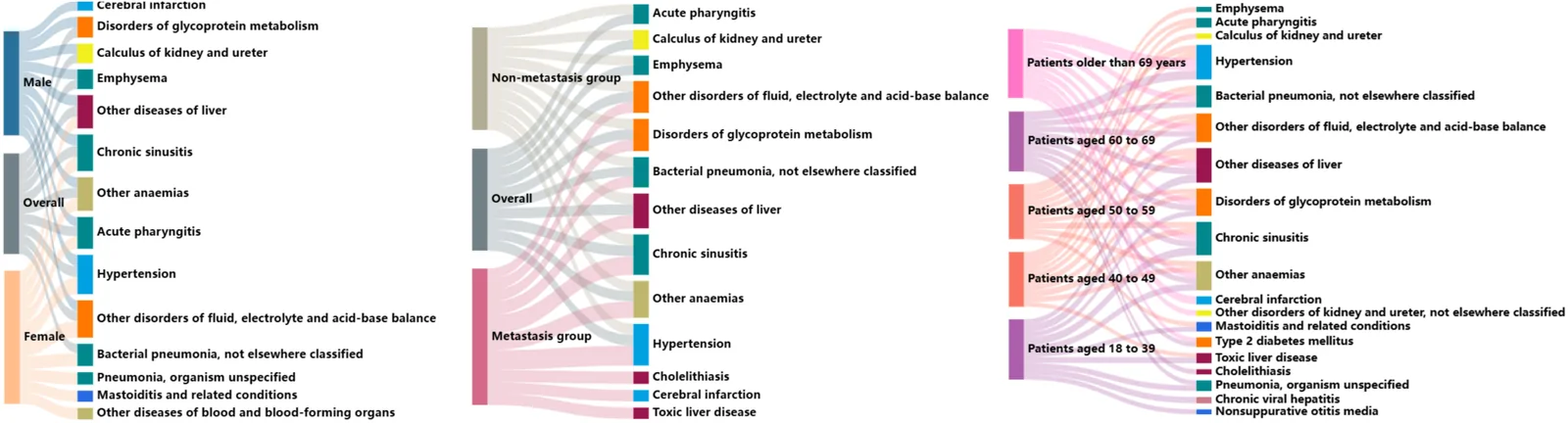
Sankey diagrams of the top 10 key comorbidities by PageRank score across NPC patient subgroups in Dataset 1. Link thickness is proportional to the PageRank value; a higher value denotes a more central and influential comorbidity within the network.

**Figure 6** shows the top 10 comorbidities ranked by PageRank score across subgroups in Dataset 1. In the overall NPC cohort of Dataset 1, hypertension (I10) and chronic sinusitis (J32) achieved the highest PageRank values at 0.028, followed by other diseases of liver (K76) at 0.027, other disorders of fluid, electrolyte and acid-base balance (E87) at 0.027, other anemias (D64) at 0.026, and disorders of glycoprotein metabolism (E77) at 0.026. For Dataset 2 overall patients, hypertension (I10) dominated the comorbidity network with a PageRank value of 0.044, followed by nonsuppurative otitis media (H65) at 0.035, chronic sinusitis (J32) at 0.034, and other anemias (D64) at 0.033. Subgroup analysis revealed notable disparities. Within Dataset 1, metastatic NPC patients exhibited the highest hypertension PageRank score at 0.051, and patients older than 69 years reached the maximum hypertension PageRank score of 0.067. In Dataset 2, hypertension attained its peak PageRank score at 0.065 among patients aged 60 to 69 years. Collectively, these comorbidities sustained elevated PageRank metrics across stratified populations, highlighting their structural prominence within the polycomorbidity network (PCN) of NPC patients.

### 3.4 PCNs for comorbidity subgroups

We stratified NPC patients into three subgroups for stratified analyses, and each subgroup included individuals diagnosed with NPC alongside one of the three most prevalent complications, namely aplastic anemia, secondary lymph node malignancies, and secondary malignancies of other organs. As shown in **Table 4**, all subgroups were male-predominant. The two secondary malignancy subgroups had consistently higher male proportions (79.1%-79.8% in Dataset 1, 78.4%-83.4% in Dataset 2) than the aplastic anemia subgroup (70.4% in Dataset 1, 69.3% in Dataset 2). The three subgroups shared similar age distributions across all age brackets. Notably, the aplastic anemia subgroup had a higher mean comorbidity burden (2.0 for both datasets) and a more dispersed comorbidity count distribution, while over half of patients in the secondary malignancy subgroups suffered from only one comorbidity in both datasets.

**Table 4:** Characteristics of nasopharyngeal carcinoma patients stratified by three complications.

Demographic and clinical factors	Dataset 1			Dataset 2		
	Aplastic anemias	Secondary malignant neoplasm of lymph nodes	Secondary malignant neoplasm of other organs	Aplastic anemias	Secondary malignant neoplasm of lymph nodes	Secondary malignant neoplasm of other organs
	(N = 2978)	(N = 2710)	(N = 3174)	(N = 1057)	(N = 597)	(N = 1024)
Male, n (%)	2097 (70.4)	2163 (79.8)	2511 (79.1)	732 (69.3)	468 (78.4)	854 (83.4)
Age (years), mean (SD)	47.2 (11.2)	47.4 (11.6)	47.9 (11.1)	47.2 (11.4)	47.7 (10.6)	48.5 (10.1)
Age group (years), n (%)						
18-39	786 (26.4)	713 (26.3)	769 (24.2)	291 (27.5)	152 (25.5)	218 (21.3)
40-49	903 (30.3)	793 (29.3)	963 (30.3)	292 (27.6)	158 (26.5)	230 (22.5)
50-59	892 (30.0)	793 (29.3)	978 (30.8)	323 (30.6)	224 (37.5)	489 (47.8)
60-69	340 (11.4)	350 (12.9)	389 (12.3)	144 (13.6)	58 (9.7)	78 (7.6)
≥70	57 (1.9)	61 (2.3)	75 (2.4)	7 (0.7)	5 (0.8)	9 (0.9)
Number of comorbidities, mean (SD)	2.0 (1.0)	1.7 (0.9)	1.7 (0.9)	2.0 (0.9)	1.4 (0.6)	1.4 (0.6)
Number of comorbidities, n (%)						
1	1127 (37.8)	1479 (54.6)	1695 (53.4)	386 (36.5)	407 (68.2)	710 (69.3)
2	1008 (33.8)	722 (26.6)	879 (27.7)	377 (35.7)	156 (26.1)	260 (25.4)
3	601 (20.2)	371 (13.7)	453 (14.3)	226 (21.4)	31 (5.2)	49 (4.8)
4	201 (6.7)	115 (4.2)	126 (4.0)	57 (5.4)	2 (0.3)	5 (0.5)
≥5	41 (1.4)	23 (0.8)	21 (0.7)	11 (1.0)	1 (0.2)	0 (0.0)

**Table 5** summarizes the patient comorbidity network (PCN) characteristics of the three subgroups. Across both datasets, the aplastic anemia subgroup exhibited a far more complex PCN structure, with higher network density, median degree, and neighbor degree than the two secondary malignant neoplasm subgroups. In Dataset 1, four conditions including other disorders of fluid, electrolyte and acid-base balance (E87), essential hypertension (I10), chronic sinusitis (J32), and other liver diseases (K76) were core shared comorbidities across all subgroups. Each subgroup had specific core comorbidities. Agranulocytosis (D70) and acute pharyngitis (J02) were exclusive to the aplastic anemia subgroup while mastoiditis and related conditions (H70) were specifically enriched in the two secondary malignancy subgroups. Pronounced dataset heterogeneity was identified as all subgroups presented markedly sparser PCNs and completely distinct core comorbidity profiles in Dataset 2 compared with Dataset 1.

**Table 5:** Phenotypic comorbidity network properties of nasopharyngeal carcinoma patients stratified by three prevalent complications in the two datasets.

Data source	Disease subgroup	Nodes, n	Density	Degree, median (IQR)	Degree of neighbors, median (IQR)	Top 10 important diseases in PCN
Dataset 1	Aplastic anemia	49	0.334	13.0( 9.0-20.0)	28.1 (24.3-30.6)	J02, E87, K76, I10, E77, J32, D75, D70, E11, N20
	Secondary malignant neoplasm of lymph nodes	50	0.279	11.0 (7.0-19.8)	22.2 (20.4-24.3)	K76, J32, I10, E87, H70, D64, E77, K71, J43, N20
	Secondary malignant neoplasm of other organs	50	0.308	13.0 (8.0-19.8)	24.1(21.6-26.8)	J32, I10, K76, D64, E87, K71, J15, H70, J43, E77
Dataset 2	Aplastic anemia	51	0.147	5.0 (3.0-9.0)	23.2 (17.6-29.5)	E87, D48, D70, K12, J34, J32, B18, H65, E77, I10
	Secondary malignant neoplasm of lymph nodes	40	0.095	2.5 (2.0-4.0)	19.3 (13.2-22.0)	D64, J15, I10, A16, N20, J98, H65, J31, E07, M10
	Secondary malignant neoplasm of other organs	46	0.075	2.0 (1.0-3.0)	25.5 (18.0-46.0)	I10, J15, G93, H65, I70, D64, B18, E87, J32, K29

## 4 Discussions

The present study examined the full spectrum of comorbidities and constructed the phenotypic comorbidity networks (PCNs) in hospitalized NPC patients diagnosed over the past 13 years in two large general hospitals located in high-incidence NPC regions. Comorbidities were prevalent at diagnosis, in which 82.9% and 78.2% of patients experienced fewer than three comorbidities. The four most prevalent comorbidities were Hypertension, chronic sinusitis, other anemias and disorders of fluid, electrolyte and acid-base balance. These conditions frequently coexisted, leading to a high comorbidity burden and increasing the complexity of disease interactions. The prevalence and coexistence patterns of comorbidities varied across subgroups categorized by sex and metastasis status. The PCN of the aplastic anemia subgroup was more complex than those of the two secondary malignant neoplasm subgroups. Although important comorbidities were similar across subgroups, each subgroup displayed distinct comorbidities.

As one of the most common malignant tumors of the head and neck (2, 3), NPC primarily influences cases aged 40 to 60 years and rarely occurs in children. The incidence is notably higher in males than in females, with a male-to-female ratio of 2–3:1 (1). Consistent with previous findings (25), the present research revealed that patients’ mean age in both datasets was approximately 47–48 years, and nearly three-quarters of NPC patients were men.

In this population-based study, 82.9% and 78.2% of patients had fewer than three comorbidities at diagnosis. A study involving 1,137 elderly NPC patients reported a 22.4% comorbidity incidence (10).The most prevalent comorbidities were hypertension, chronic sinusitis, other anemias and disorders of fluid, electrolyte and acid-base balance in the present study. Physiological aging and prevalent unhealthy lifestyles, including smoking and excessive drinking, result in a high prevalence of hypertension in middle-aged and elderly populations (26, 27), []which explains why hypertension is the most common pre-existing chronic disease among NPC patients. The anatomical adjacency between the nasopharynx and paranasal sinuses causes chronic sinusitis to interact mutually with NPC lesions (28). Other anemias are frequently detected during the diagnosis and treatment of NPC patients, which may be associated with tumor-related chronic inflammation, inadequate nutritional intake, and chemoradiotherapy-induced myelosuppression. Tumor consumption, treatment-related gastrointestinal adverse reactions, recurrent vomiting and impaired renal regulatory function may predispose NPC patients to disorders of fluid, electrolyte and acid-base balance. Comorbidities in NPC patients link to multiple factors, including the tumor’s biological characteristics, treatment-related side effects, viral infections, age-related vulnerabilities, and pre-existing diseases.

In the PCNs of NPC patients in this study, Hypertension, chronic sinusitis and other anemias were core high-prevalence hub conditions in both networks, and their strong inter-disease connections substantially increased multimorbidity complexity. Similar findings have been reported, though the comorbidity prevalence varied (8, 10). Disease connections can be direct or indirect. Direct correlations are speculated to stem from shared underlying pathogenic pathways, including chronic systemic inflammation, EBV-related immune dysfunction, and metabolic dysregulation, which may collectively facilitate the co-occurrence of multiple comorbidities among NPC patients. For example, sustained inflammation triggered by EBV infection and tumor progression may plausibly exacerbate local sinus inflammation and hematopoietic abnormalities, while also contributing to vascular endothelial injury and thereby elevating the risk of hypertension. Indirect correlations are presumably mediated by external confounding factors, such as advanced age, unhealthy lifestyle habits, and potential chemoradiotherapy-related systemic toxicities (26). Nevertheless, these interpretations remain speculative, as individual treatment records and dynamic laboratory indicators were insufficient to verify the direct effects of treatment toxicity or viral immune disturbance on comorbidity progression in this retrospective cohort. The present study also found that respiratory diseases acted as the primary mediators of comorbidity linkages across both cohorts. This suggests that these comorbidities mediate correlations among various other comorbidities associated with NPC. Collectively, these hub comorbidities do not exist in isolation, and their correlated interactions may jointly aggravate the physical burden of NPC patients. These correlational network features might partially explain the prognostic heterogeneity of NPC patients with multimorbidity, without confirming causal mechanisms.

Subgroup differences show males have more comorbidities than females in NPC, regardless of age. Three-quarters of patients were male, possibly causing sex bias in this study. Biological differences between males and females, as well as lifestyle factors (e.g., smoking and alcohol consumption, both associated with the development of multiple coexisting conditions), may increase the complexity of managing cancer and its comorbidities. This may also account for the more complex comorbidity coexistence relationships in males compared with females. In addition, we also found that males had higher prevalence of chronic viral hepatitis and calculus of kidney and ureter, while females had more fluid, electrolyte, acid-base disorders. In the era of precision medicine, considering sex differences in assessing disease development and prognosis is increasingly noteworthy (29).

In this study, the average number of comorbidities was higher in the non-metastasis subgroup than in the metastasis subgroup, and non-metastatic NPC patients exhibited more complex comorbidity connections in PCN analysis. Non-metastatic NPC patients are predominantly diagnosed with early-to-middle stage disease, with relatively prolonged survival time and stable physical status (30). These patients tend to retain long-term exposure to age-related physiological decline, chronic inflammatory conditions, and unhealthy lifestyle risk factors, which may continuously accumulate and induce multiple chronic comorbidities over the disease course. The more intricate comorbidity connections in non-metastatic subgroups further suggest that multimorbidity interactions are more abundant and sophisticated in early-stage NPC patients with chronic disease accumulation. Overall, these findings indicate that the comorbidity burden and network complexity of NPC patients cannot be simply inferred by tumor metastatic status. This study provides novel evidence for individualized comorbidity management, emphasizing that early-stage non-metastatic NPC patients also require systematic multimorbidity screening and targeted intervention to reduce long-term health risks.

Regarding the age of NPC patients in this study, the mean number of comorbidities increased with age in both males and females under 60 years old, which was consistent with previous studies (31). Certain comorbidities, particularly hypertension, exhibited a notable increase with advancing age. In addition, comorbidity counts tended to rise progressively with advancing age in NPC patients, irrespective of tumor metastasis. For these patients, comprehensive management strategies are essential to control hypertension and other comorbidities. These strategies include regular blood pressure monitoring, promoting a healthy lifestyle, and administering medication when necessary (8).

In studies categorizing the three most prevalent comorbid complications into distinct subgroups, the PCN of the aplastic anemia subgroup was more complex than those of the two secondary malignant neoplasm subgroups. This complexity was reflected in higher network density, degree, and neighbor degree. The inherent characteristics of aplastic anemia may contribute to widespread immune dysfunction and an elevated infections risk (32). The management and treatment of these complications further increase the network’s complexity. Though secondary malignancies bring complexities, their comorbidity networks are relatively concentrated and closely tied to treatment factors. Noteworthy, the important comorbidities were similar among the three subgroups. However, the aplastic anemia subgroup exhibited two unique comorbidities, including agranulocytosis and acute pharyngitis. This may relate to impaired bone marrow hematopoiesis, immune dysfunction, and microenvironment changes or genetic mutations in these patients (33, 34). Conversely, patients with secondary malignancy were more prone to developing mastoiditis and related conditions.

Additionally, marked non-random disparities in metastasis and multimorbidity proportions exist between the two datasets, which may be attributed to the distinct patient compositions of the two hospitals. Dataset 1 mainly includes newly diagnosed early-stage NPC patients with a metastasis rate of only 3.5%, while Dataset 2 admits large numbers of transferred advanced metastatic patients, leading to a much higher metastasis rate of 40.8%. This difference in patient sources accounts for such prominent numerical gaps. Despite these numerical gaps, consistent comorbidity network and clustering trends were observed in both cohorts, confirming the stability of our core findings. However, caution should be exercised when generalizing these quantitative prevalence results to other medical centers.

This study has some limitations. Firstly, the absence of survival analysis and in-depth multi-omics/clinical data discussions for k-means cluster-defined populations is a significant constraint. As a retrospective study with a large dataset, critical clinical information, such as specific death times, laboratory indicators, and medication records was missing, and our current database lacks the functionality to query or export these details. Restricted by the research design and electronic medical record data, we could not obtain complete treatment archives to validate causal links between radiotherapy/chemotherapy toxicity and multimorbidity, nor correlate disease clusters with patient-level outcomes. This data gap restricts our ability to fully explore the prognostic and clinical relevance of the identified clusters. Radiomics and machine learning have been widely applied in the prediction and evaluation of the therapeutic effect of nasopharyngeal carcinoma (35, 36). With the development of artificial intelligence (AI) and the establishment of a complete database, we hope to complete further in-depth research with the help of AI in the future. Secondly, while it identified comorbidity patterns specific to NPC patients by subgroup, it did not compare these patterns with those observed in other cancers. Furthermore, the study was limited to a single high-incidence region; the lack of data from low-incidence areas may limit the generalizability of the findings. Future research should expand this by comparing NPC with other malignancies and include cases from diverse regions to better understand its unique comorbidity landscape. Thirdly, although NPC treatment guidelines have become more standardized, variations in treatment approaches and quality of management still exist across hospitals. As cases in this study were recruited from multiple hospitals without concentration on treatment protocols, this could introduce potential variability in the results. Additionally, the extreme sample size imbalance between metastatic and non-metastatic subgroups in Dataset 1 (with metastatic patients accounting for merely 3.5%) is an inherent limitation of real-world data. Network topological indicators are susceptible to variations in sample volume, which may impair direct cross-group comparisons of comorbidity networks. Multi-center prospective studies will be conducted to recruit more patients with metastatic NPC and achieve a more balanced case distribution between subgroups in future research.

## 5 Conclusions

The present study indicated significant differences in the prevalence of comorbidities and their complex coexistence between male and female patients, as well as between metastatic and non-metastatic subgroups of NPC patients. The presence of these comorbidities not only complicates treatment, but also impacts disease prognosis. Therefore, the management of NPC patients should comprehensively address these comorbidities and implement appropriate preventive and therapeutic measures. These measures include lifestyle modifications, regular screening and monitoring, and employing a combination of treatment modalities, aiming to improve the treatment effect and quality of life of NPC patients.

## Data Availability

The original contributions presented in the study are included in the article/**Supplementary Material**. Further inquiries can be directed to the corresponding authors.
