# Supplementary material for: Phenotypic comorbidity network analysis of nasopharyngeal carcinoma: a multicenter study based on hospital discharge records
Source: Front Oncol. 2026 Sep 10;16:1895769. doi: 10.3389/fonc.2026.1895769 (PMC13600998; doi:10.3389/fonc.2026.1895769)
Supplement: Supplementary file 1 [file DataSheet1.docx]

Supplementary Material

**Figure S1.** Age distribution (A, D), comorbidity count distribution (B, E), and mean comorbidity burden by age group (C, F) in nasopharyngeal cancer patients from Dataset 2, with A-C stratified by sex and D-F as their counterparts stratified by metastasis status.

# Table S1. Disease codes and diseases names.

| **ICD-10 code** | **Disease name** |
| --- | --- |
| A16 | Respiratory tuberculosis, not confirmed bacteriologically or histologically |
| B02 | Zoster [herpes zoster] |
| B18 | Chronic viral hepatitis |
| B66 | Other fluke infections |
| B90 | Sequelae of tuberculosis |
| C11 | Malignant neoplasm of nasopharynx |
| C77 | Secondary and unspecified malignant neoplasm of lymph nodes |
| C78 | Secondary malignant neoplasm of respiratory and digestive organs |
| C79 | Secondary malignant neoplasm of other and unspecified sites |
| C80 | Malignant neoplasm, without specification of site |
| C97 | Malignant neoplasms of independent (primary) multiple sites |
| D18 | Haemangioma and lymphangioma, any site |
| D48 | Neoplasm of uncertain or unknown behaviour of other and unspecified sites |
| D61 | Other aplastic anaemias |
| D63 | Anaemia in chronic diseases classified elsewhere |
| D64 | Other anaemias |
| D69 | Purpura and other haemorrhagic conditions |
| D70 | Agranulocytosis |
| D75 | Other diseases of blood and blood-forming organs |
| D86 | Sarcoidosis |
| E03 | Other hypothyroidism |
| E04 | Other nontoxic goitre |
| E07 | Other disorders of thyroid |
| E11 | Type 2 diabetes mellitus |
| E14 | Unspecified diabetes mellitus |
| E46 | Unspecified protein-energy malnutrition |
| E77 | Disorders of glycoprotein metabolism |
| E78 | Disorders of lipoprotein metabolism and other lipidaemias |
| E79 | Disorders of purine and pyrimidine metabolism |
| E87 | Other disorders of fluid, electrolyte and acid-base balance |
| F41 | Other anxiety disorders |
| G93 | Other disorders of brain |
| H35 | Other retinal disorders |
| H65 | Nonsuppurative otitis media |
| H66 | Suppurative and unspecified otitis media |
| H70 | Mastoiditis and related conditions |
| H90 | Conductive and sensorineural hearing loss |
| I10 | Essential (primary) hypertension |
| I25 | Chronic ischaemic heart disease |
| I44 | Atrioventricular and left bundle-branch block |
| I49 | Other cardiac arrhythmias |
| I63 | Cerebral infarction |
| I70 | Atherosclerosis |
| J02 | Acute pharyngitis |
| J03 | Acute tonsillitis |
| J06 | Acute upper respiratory infections of multiple and unspecified sites |
| J15 | Bacterial pneumonia, not elsewhere classified |
| J18 | Pneumonia, organism unspecified |
| J31 | Chronic rhinitis, nasopharyngitis and pharyngitis |
| J32 | Chronic sinusitis |
| J34 | Other disorders of nose and nasal sinuses |
| J38 | Diseases of vocal cords and larynx, not elsewhere classified |
| J39 | Other diseases of upper respiratory tract |
| J43 | Emphysema |
| J47 | Bronchiectasis |
| J98 | Other respiratory disorders |
| K11 | Diseases of salivary glands |
| K12 | Stomatitis and related lesions |
| K29 | Gastritis and duodenitis |
| K71 | Toxic liver disease |
| K74 | Fibrosis and cirrhosis of liver |
| K76 | Other diseases of liver |
| K77 | Liver disorders in diseases classified elsewhere |
| K80 | Cholelithiasis |
| K82 | Other diseases of gallbladder |
| L58 | Radiodermatitis |
| M10 | Gout |
| M33 | Dermatopolymyositis |
| M48 | Other spondylopathies |
| M84 | Disorders of continuity of bone |
| M89 | Other disorders of bone |
| N19 | Unspecified kidney failure |
| N20 | Calculus of kidney and ureter |
| N28 | Other disorders of kidney and ureter, not elsewhere classified |
| N40 | Hyperplasia of prostate |
